# Policies and Programs to Facilitate Access to Targeted Cancer Therapies in Thailand

**DOI:** 10.1371/journal.pone.0119945

**Published:** 2015-03-23

**Authors:** Rosarin Sruamsiri, Dennis Ross-Degnan, Christine Y. Lu, Nathorn Chaiyakunapruk, Anita K. Wagner

**Affiliations:** 1 Center of Pharmaceutical Outcomes Research, Department of Pharmacy Practice, Faculty of Pharmaceutical Sciences, Naresuan University, Phitsanulok, Thailand; 2 Department of Population Medicine, Harvard Medical School and Harvard Pilgrim Health Care Institute, Boston, Massachusetts, United States of America; 3 School of Pharmacy, Monash University Malaysia, Malaysia; 4 School of Population Health, University of Queensland, Brisbane, Australia; 5 School of Pharmacy, University of Wisconsin-Madison, Madison, WI, United States of America; Mario Negri Institute for Pharmacology Research, ITALY

## Abstract

**Background:**

Increasing access to clinically beneficial targeted cancer medicines is a challenge in every country due to their high cost. We describe the interplay of innovative policies and programs involving multiple stakeholders to facilitate access to these medicines in Thailand, as well as the utilization of selected targeted therapies over time.

**Methods:**

We selected two medicines on the 2013 Thai national list of essential medicines (NLEM) [letrozole and imatinib] and three unlisted medicines for the same indications [trastuzumab, nilotinib and dasatinib]. We created timelines of access policies and programs for these products based on scientific and grey literature. Using IMS Health sales data, we described the trajectories of sales volumes of the study medicines between January 2001 and December 2012. We compared estimated average numbers of patients treated before and after the implementation of policies and programs for each product.

**Results:**

Different stakeholders implemented multiple interventions to increase access to the study medicines for different patient populations. During 2007–2009, the Thai Government created a special NLEM category with different coverage requirements for payers and issued compulsory licenses; payers negotiated prices with manufacturers and engaged in pooled procurement; pharmaceutical companies expanded patient assistance programs and lowered prices in different ways. Compared to before the interventions, estimated numbers of patients treated with each medicine increased significantly afterwards: for letrozole from 645 (95% CI 366–923) to 3683 (95% CI 2,748–4,618); for imatinib from 103 (95% CI 72–174) to 350 (95% CI 307–398); and for trastuzumab from 68 (95% CI 45–118) to 412 (95% CI 344–563).

**Conclusions:**

Government, payers, and manufacturers implemented multi-pronged approaches to facilitate access to targeted cancer therapies for the Thai population, which differed by medicine. Routine monitoring is needed to assess clinical and economic impacts of these strategies in the health system.

## Introduction

Cancer constitutes a major disease burden especially in low- and middle-income countries (LMICs), where most health systems are ill-prepared to meet the challenges of providing and financing cancer care.[1,2,3] More than half of the 12.4 million new cases of cancer in 2008 and almost two-thirds of the annual 7.6 million deaths from cancer occurred in LMICs.[4,5] By 2030, an estimated 27 million new cancer cases and 17 million cancer deaths will occur in LMICs.[2] Cancer impacts the health and emotions of patients and families, and the costs of cancer care can impoverish both families and health systems.

Cancer survival tends to be poorer in developing countries than developed ones. In developed countries, cancer treatment is a priority and well-functioning health care infrastructures often facilitate earlier cancer detection, better access to treatment, and reduction of mortality.[6] With limited resources for health, LMICs struggle to guarantee access for all members of society to needed cancer treatments, especially innovative but expensive cancer medicines.[7,8] Targeted cancer therapies which act on specific molecules involved in tumor growth and progression play a significant role in modern cancer care; new medicines are estimated to account for 50–60 percent of the increase in cancer survival rates in high-income countries since 1975.[9] Due to the high costs of these innovative medicines, many cancer patients, particularly in LMICs, face substantial financial barriers to accessing promising treatments.

Many countries are striving toward universal health coverage (UHC), intending to ensure that everyone has access to needed health services without incurring financial hardship.[10,11] UHC proponents aim to overcome major inequalities in access to care, inadequate risk protection, poor affordability of health services, and high household out-of-pocket health expenditures.[7] While countries may embark on different paths toward UHC,[12] all policy makers face resource constraints and competing health priorities when making difficult decisions about providing access to innovative cancer medicines for their populations. Governments frequently use health technology assessment (HTA) to decide which cancer medicines to list as essential medicines and to cover through insurance or by public subsidy systems. Pharmaceutical companies may also facilitate access by lowering prices and providing free medicines to specific patients. Evidence is needed on the effects of different strategies that facilitate access to innovative cancer medicines in systems working toward UHC.

In Thailand, an upper-middle income country in South-East Asia, cancer is a leading cause of death.[13,14] From 2003 to 2011, the mortality rate from cancer rose from 79 to 95 per 100,000 population.[13] Thailand implemented a comprehensive strategy toward universal health coverage in 2002, after which all Thais were covered by health insurance guaranteeing access to a comprehensive package of health services.[15] In 2013, the Civil Servant Medical Benefit Scheme (CSMBS) for government employees covered 7.6 percent of the population,[16] the Social Security Scheme (SSS) for private sector employees another 16.1 percent, and the rest of the population (76.3 percent) was enrolled in the Universal Coverage (UC) Scheme,[16] which covers everyone regardless of socioeconomic status. Member contributions, medical and pharmacy benefits, and reimbursement procedures differ between the three schemes.[17] For members of the UC and SSS schemes, medicines listed on the National List of Essential Medicines (NLEM) are covered under a capitated benefit when prescribed during ambulatory care, or under case-based reimbursement when prescribed for and administered to inpatients. UC and SSS members need to pay out of pocket for medicines not listed on the NLEM. For CSMBS patients, providers receive fee-for-service payments; most NLEM and non-NLEM medicines are free of charge for CSMBS members.

Since the health care reform in 2001,[18,19] several stakeholders have initiated policies and programs to facilitate access to medicines. The Thai government implemented an evidence-based process for selecting medicines for the NLEM, established a minimum medicines reimbursement list, exercised the World Trade Organization (WTO) Trade-Related Aspects of Intellectual Property Rights (TRIPs) provisions for several medicines,[20,21] and adopted HTA for making decisions about whether new technologies should be added to the NLEM.[13]

In 1981, the Thai government issued its first NLEM.[22] The tenth version of the NLEM, published in September, 2013,[23] lists 832 medicines considered essential for treating the health problems of Thai people.[2] The list is the basis for medicines procurement by public hospitals and constitutes the minimum reimbursement list for all major health insurance schemes. Medicines are selected based on whether they meet health needs, as well as safety, efficacy, quality, cost-effectiveness and national affordability criteria.[20] For innovative and high cost medicines deemed important, the NLEM Committee also conducts HTAs to determine their cost-effectiveness and budget impact to support NLEM decision making.[20,24] Currently, the NLEM comprises five major categories of medicines based on indications, appropriateness, and prescribing requirements: categories A-C comprise the medicines necessary to treat common diseases at every level of hospital; category D contains medicines that may be life-saving for some patients[2] but require expert diagnosis before prescribing; and category E contains specialized medicines that require pre-approval and patient laboratory monitoring. A limited number of innovative cancer medicines have been included in the NLEM since 1981: of 26 targeted cancer therapies approved in Thailand, four are listed in the 2013 NLEM.[2,25] In addition, the Thai government has also implemented compulsory licensing (CL) under TRIPs; the Thai Ministry of Public Health granted CLs for four patented cancer drugs in January, 2008: letrozole for breast cancer, docetaxel for breast and lung cancers, erlotinib for lung cancer, and imatinib for chronic myelocytic leukemia (CML) and gastrointestinal stromal tumour (GIST).[26,27] Compulsory licenses, when implemented, allow the government to produce or import less expensive generic versions of the respective drugs without agreement of the patent holder, However, before the implementation of CL for cancer drugs products, the government allowed patent-owners to lower prices of the respective branded products, in lieu of issuing a CL.

Other stakeholders have also implemented programs to increase access to innovative cancer medicines. Through patient assistance programs, companies collaborate with non-government organisations (NGOs) to provide free medicines to selected individual patients.[28] Government suppliers pool purchasing of bulk volumes and negotiate special purchasing arrangements with companies resulting in lower prices.

Thailand’s experiences may inform other countries on the path to UHC. However, no systematic assessment exists of the impacts of strategies implemented by different stakeholders in Thailand to increase access to innovative cancer therapies. Thus, the objectives of the present study were to: a) describe the policy and program approaches by different health system stakeholders in Thailand to facilitate access to targeted cancer therapies; and b) analyze utilization of selected targeted cancer treatments over time.

## Methods

### Selection of targeted therapies

Ethical approval was granted by the IRB of Harvard Pilgrim Health Care Institute. Using IMS Health data (described below) [9, 10], we identified all cancer treatments from three therapeutic classes [antineoplastics, immunostimulating agents, and cytostatic hormones, as classified by the European Pharmaceutical Research Association Anatomical Therapeutic Chemical (ATC) system] on the market in Thailand in 2012 (n = 107 different molecules). Of 76 cancer medicines launched before 2001, seven (9%) were targeted therapies, versus 19 (60%) of 31 launched during 2001–2012.

Four targeted therapies [two launched before 2001 (basiliximab and letrozole) and two launched during 2001–2012 (imatinib and bevacizumab)] were listed in the 2013 NLEM.[2] We selected two listed products: letrozole, which is indicated for breast cancer, the most common cancer in Thailand;[29] and imatinib, which is indicated for the treatment of Philadelphia chromosome positive (CML-Ph+) chronic myelogenous leukemia and gastrointestinal stromal tumors (GISTs). To illustrate policy differences, we also selected three targeted cancer therapies used for the same indications but not listed in the NLEM: trastuzumab for breast cancer with positive human epidermal growth factor receptor 2 (HER2+), and nilotinib and dasatinib for treatment of CML and GISTs. [40–42]

### Policy and program identification and analysis

We identified policies and programs intended to increase access to the study medicines in Thailand in multiple ways. We searched electronic databases including PubMed[30] through December 2013 for published articles on Thai policy changes related to cancer medicines. We also searched for published policy documents on the websites of the Ministry of Public Health and the three health insurance schemes (the National Health Security Office (NHSO) in charge of the UC scheme,[31] the Social Welfare Office (SWO) in charge of the SSS,[32] and the Comptroller General’s Department, Ministry of Finance (CGD) in charge of the CSMBS[33]). In addition, we searched for relevant information on research institute websites, the Open University website, and the websites of the pharmaceutical companies. Search terms included: (“policy” OR “intervention” OR “program” OR “patient access program” OR “patient assistance program”) AND “access to medicines” AND (“targeted cancer therapies” OR “letrozole” OR “trastuzumab” OR “imatinib” OR “nilotinib” OR “dasatinib”) AND “Thailand”. Relevant policies and programs implemented during 2001–2012 were included.

We categorized policies and programs into three groups, according to the stakeholders who initiated them: the central government, payers, and pharmaceutical companies. We verified policy details in personal communications with selected stakeholders.

### Analysis of medication use

We assessed the utilization of targeted cancer therapies using quarterly IMS Health sales data from 2001 to 2012.[34] IMS Health data originate from pharmaceutical companies’ sales reports and surveys of purchases by 200 of the 1100 public and private hospitals in Thailand; the data are projected to represent total national sales. More than 85% of cancer drugs’ sales occur in the public sector. [35] Information on medicines dispensed in public and private hospitals under special programs initiated by pharmaceutical companies or non-governmental organizations is included in the estimates. The database contains generic drug names, product names, pack sizes, and volumes sold in standard units. A standard unit is defined by IMS Health as the smallest unit of a dosage form, that is, one tablet or capsule for oral products and one vial for injections. To account for population changes, we used semi-annual population estimates from the National Statistical Office as the denominator in our analyses.[36] We depict semiannual time series of the number of standard units sold per 100,000 people, assuming that increasing sales volumes represent increasing access to the medicines.

We estimated the number of patients treated in a given half-year period as the total number of courses of each targeted therapy sold divided by standard adult dose for a specific indication. To do so, we transformed standard units into total amount of drug (in milligrams) sold and divided drug amount by recommended standard doses for each indication. We used recommended doses based on local clinical practice guidelines. For letrozole and imatinib, we applied recommended doses from the 2013 NLEM [23] and obtained recommended doses for non-NLEM medicines (dasatinib nilotinib and trastuzumab) from local clinical practice guidelines.[37,38,39] Dosage assumptions are listed in Table 1: letrozole 2.5 mg per day for metastatic breast cancer in postmenopausal patients with positive hormone receptor; trastuzumab loading dose 4 mg/kg and maintenance dose 2 mg/kg every week, assuming a standard adult women body weight of 57 kilogram,[40] for treatment of HER2+ metastatic breast cancer patients; imatinib 400 mg, dasatinib 100 mg and nilotinib 800 mg per day for CML-Ph+ and GISTs.[41,42] We also assumed that patients received all treatments as detailed in the guidelines and that all medicines were used as indicated. Using a paired Mann-Whitney U Test, we compared median estimated numbers of patients treated across the half-year periods before to those after the sets of policies were implemented for each targeted therapy. We calculated confidence intervals around estimated median numbers of patients treated.

**Table 1:** Standard treatment of targeted cancer therapy.

Targeted cancer therapy	Daily dose (mg)	Treatment duration	Total amount per case per quarter (mg)
Letrozole	2.5	Once daily	227.5
Trastuzumab	Loading dose: 8 mg/kg	Once	2,964*
	Maintainance dose: 4 mg/kg	Every week	
Imatinib	400	Once daily	36,400
Nilotinib	800	Once daily	72,800
Dasatinib	100	Once daily	9,100

* Calculation based on a standard adult women body weight of 57 kilogram

## Results

**Fig 1:**
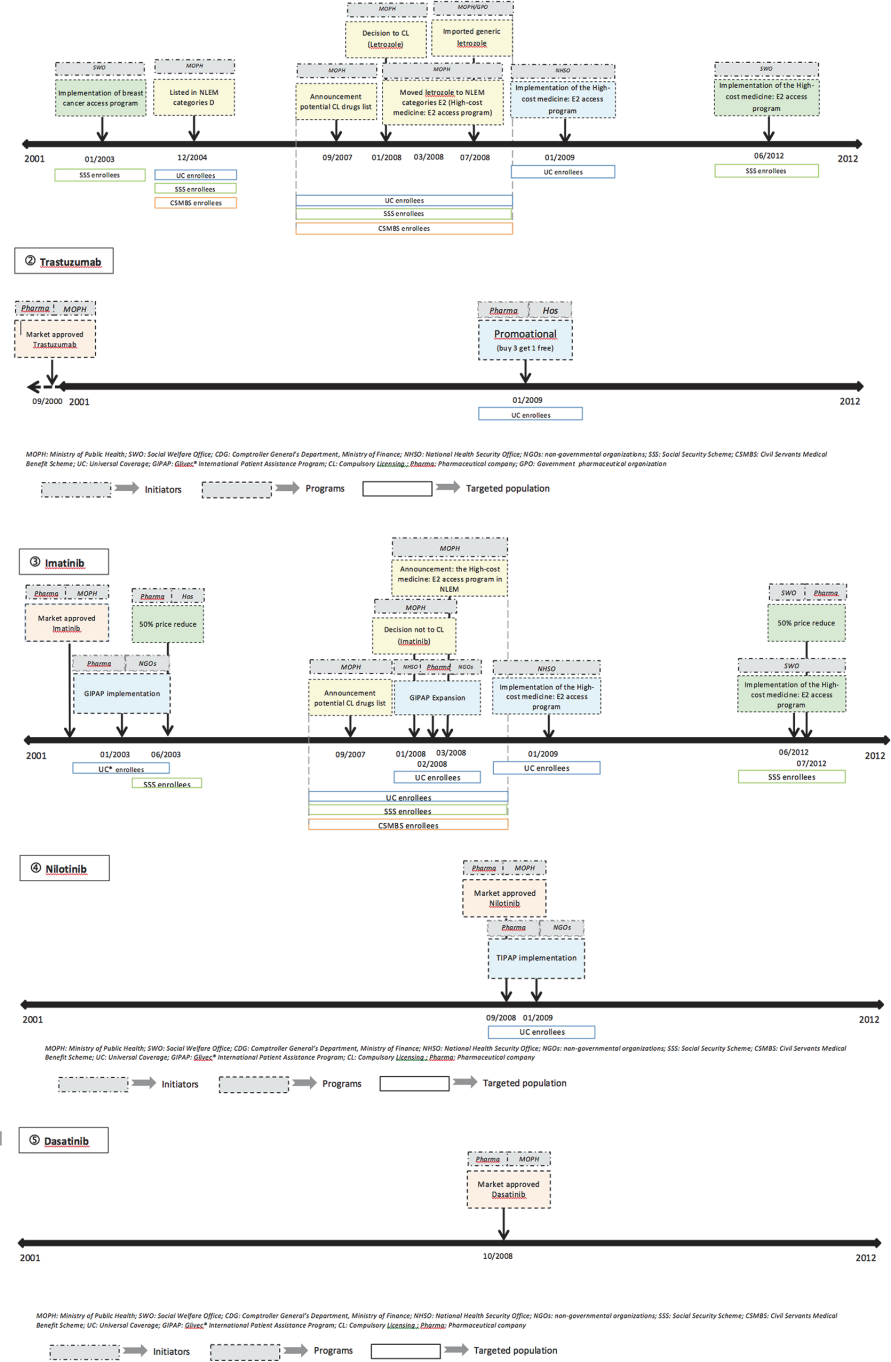
Chronology of government, payer, and manufacturer policies facilitating patient access to five targeted cancer therapies.

Published literature [20,26,28,43] documented four policies intended to improve access to the study medicines; threes additional policies were described in local documents. Policies are summarized in Table 2. The government, insurance payers and pharmaceutical companies had each initiated several policies and programs, creating a complex and changing policy environment around targeted cancer therapies. Fig. 1 presents chronological timelines of the sequence of policy approaches over time for each medicine. Below we describe the policy and utilization changes for each selected medicine.

**Table 2:** Summary of policies.

Initiators	Policies
**Government**	○ National List of Essential Medicines (NLEM)
	○ E2 access program implementation
	○ Compulsory licenses (CL)
**Payers**	
National Health Security Office (NHSO)	○ E2 access program implementation
	○ Pooled purchasing (price negotiation)
	○ Medicine reimbursement policies
Social Welfare Office (SWO)	○ E2 access program implementation
	○ Special marketing arrangements (price negotiation)
Comptroller General’s Department (CGD)	○ E2 access program implementation
**Pharmaceutical companies**	○ Patient assistance programs
	○ Special marketing arrangements (price negotiation)

### Letrozole (NLEM listed)

#### Policy and program approaches

Government-initiated access policies: To facilitate access to letrozole, in 2004 the government included letrozole in NLEM category D (“medicines that are used for specific indications[2] and require specialist physician diagnosis and monitoring”) for postmenopausal women with hormone-receptor-positive breast cancer.[44] Due to its high cost (approximately US$2555 per year in 2012, about 25 times the fixed capitation rate of hospitals for SSS and UC patients[45]), letrozole was moved from NLEM category D to sub-category E2 (“High-cost medicines: E2 access program”) that was first created in the 2008 NLEM.[46,47] The E2 access program was established for very high cost medicines considered to be an economic burden to both society and patients[33] and for which appropriate prescribing is limited to strictly defined clinical situations. To ensure access and appropriate use, the E2 access program requires that insurers provide medicines in the NLEM E2 category free of charge to patients who meet prior authorization requirements and for whom the products are prescribed by qualified physicians. Different from other NLEM category drugs, insurance schemes were given time to implement the E2 access program so that they could allocate resources to cover the medicines. NHSO implemented the E2 policy for letrozole in January 2009; the SWO implemented it in June 2012.

Because of ongoing concerns about the cost of letrozole, which limited patients’ access to the medicine, the Thai government considered exercising its WTO TRIPs flexibilities to issue a CL for letrozole.[25,48,49,50] In September 2007, the Government granted a series of CLs to allow the import of generic equivalents of four patented cancer medicines (i.e., imatinib, letrozole, docetaxel and erlotinib) into Thailand. However, beginning mid-October, 2007, the Public Health Minister decided to negotiate prices with patent-holding pharmaceutical companies to encourage them to expand access to their cancer drugs, possibly in lieu of a CL.[25,32] After several rounds of negotiations, the Thai government finally implemented a CL for letrozole in January 2008. Since no generic letrozole was available in the Thai market at the time, the government imported generic letrozole from India in July 2008.[25]

Payer-initiated access policies: 1) The National Health Security Office: Although letrozole had been listed on NLEM since 2004, due to its high cost, limited numbers of UC patients received the drug. Both NHSO and hospitals could not afford the expenditures. The implementation of a CL in September 2007 and the E2 access program in March 2008 were intended to facilitate access. NHSO took nine months to prepare their system and became the first payer to implement the E2 access program in January 2009.[46] All postmenopausal women with hormone-receptor-positive metastatic breast cancer enrolled in UC began to receive letrozole free of charge. For stock management and to ensure continuous supply to hospitals, NHSO worked with the Government Pharmaceutical Organization (GPO)[51] to procure generic letrozole for use by UC patients. GPO procures generic products from India under its aggregate medicines procurement plan and supplies each hospital according to demand under its Vendor Managed Inventory (VMI) system.[46,52] 2) Social Welfare Office: Discrepancies in medical benefits between patients under UC and SSS were identified after the NHSO implemented the E2 access program.[46] SWO patients needed to pay for target medicines because the SWO had not yet implemented the program for SSS enrollees. After a long period of preparation, the SWO implemented the E2 access program in June 2012.[53] 3) Comptroller General’s Department, Ministry of Finance (CDG): No special access policy or program was initiated for CSMBS enrollees because letrozole was already covered under their fee-for-service health and pharmacy benefits.

#### Utilization over time

**Fig 2:**
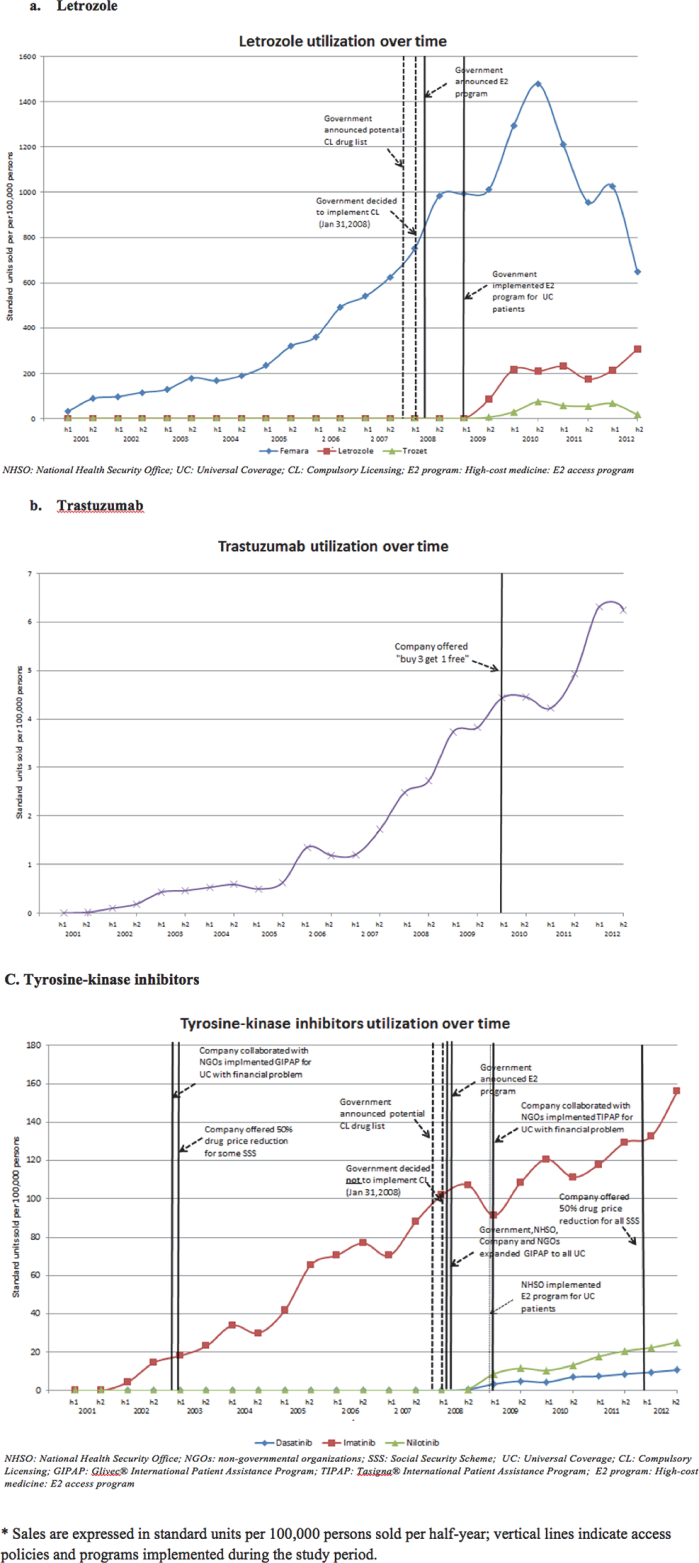
Sales of selected targeted cancer therapies over time.

Fig. 2(A) shows the utilization of branded and generic letrozole over time. Based on the standard dosage of letrozole (Table 1), during the observation period, the median estimated number of patients treated with either branded or generic letrozole increased from 645 (95% CI 366–923) in the half-year periods before to 3683 (95% CI 2748–4618) in those after the 2009 implementation of the access policies.

Between 2001 and 2008, only branded letrozole existed in the market and its use increased steadily and substantially, from 160 to 2232 patients treated. After implementation of CL and the E2 access program, sales per half-year per 100,000 persons of originator-brand letrozole remained initially stable. Sales of two generic letrozole products (Letrozole and Trozet) that entered the market in 2009 following implementation of CL rose quickly to about 200 and 50 standard units sold/100,000 persons, respectively. However, a shortage of generic letrozole imported from India in the second half of 2010 prompted the NHSO to procure letrozole from the originator company Novartis to guarantee availability at hospitals, accounting for the sudden 2010 peak in originator-brand letrozole (Femara) sales of about 1500 units/100,000 persons.

### Trastuzumab (Non-NLEM)

#### Policy and program approaches

Trastuzumab was approved for the treatment of metastatic HER2+ breast cancer in Thailand in September 2000.[3] However, as of May 2014, trastuzumab had not been listed on the NLEM.[2,33] Only patients covered by the CSMBS had access the medicine free of charge; SSS and UC enrollees needed to pay out of pocket for trastuzumab treatment. The manufacturer submitted an application for NLEM listing in 2010 and the NLEM committee is waiting for HTA results from the Health Economics Working Group and a price proposed by the Price Negotiation for NLEM Selection Working Group.[20]

Pharmaceutical company initiated special marketing arrangements: To facilitate access to trastuzumab, Roche Co. Ltd began to offer in January 2010 a program (“Buy 3 and get 1 free”) that decreased treatment costs for self-paying patients while waiting for NLEM committee decisions.[54]

#### Utilization over time

Fig. 2(B) shows the growth in sales of trastuzumab over time. The sales of trastuzumab increased slightly between 2001 and 2007, and started to steeply increase in 2006. After implementation of the company-initiated patient assistance program in 2010, trastuzumab sales may have increased somewhat more steeply starting in 2011. Assuming that trastuzumab was used only for metastatic breast cancer (the only CSMBS-reimbursed indication for this drug) and using standard doses for this indication (Table 1), the median estimated number of patients treated per half year during 2001–2009 was 136 (95% CI 90–238); and 824 (95% CI 688–1,126) between 2010 and 2013.

### Imatinib (NLEM listed)

#### Policy and program approaches

Imatinib was the first tyrosine kinase inhibitor approved for the treatment of CML in 2002 and for GISTs in 2003. Government authorities, payers and the manufacturer have introduced policies and programs to facilitate access to imatinib.

Government initiated access policies: The Thai government-issued CLs in September 2007 for four cancer drugs included imatinib. However, Novartis, the manufacturer of imatinib, made a last minute offer to the Thai government to provide the medicine free to all patients under the UC scheme in January 2008, provided that no CL would be issued.[50]

Similar to letrozole, imatinib was listed in March 2008 in the E2 program under the NLEM for patients with CML-Ph+ and GISTs.[46] However, payment and distribution of imatinib for UC-covered patients remained under GIPAP (described below).

Payer initiated access policies: Since February 2008, selected CML patients covered by the UC scheme received imatinib free of charge under the Glivec International Patient Assistance Program (GIPAP). Some patients covered under the SSS received imatinib for half the original price, following price negotiations between the company and selected hospitals that engaged in negotiations. The different payers then implemented the imatinib E2 access program at different times, the NHSO in January 2009 and the SWO in June 2012. While approaches differed by scheme, they involved agreements with the manufacturer Novartis described below.

Pharmaceutical company initiated access programs: GIPAP had been set up in Thailand since January 2003 by Novartis Co Ltd. to facilitate access to and distribution of imatinib directly to CML patients though registered hospitals. The program aimed to fill the gaps in access for eligible patients who could not afford the costly treatment.[55] Under the program, patients must be diagnosed by a GIPAP-qualified physician. Physicians submit an application on behalf of each patient. The physician is required to be involved in all stages of treatment, including diagnosis, prescription, and follow-up. Patient applications are assessed by the Max Foundation, a US-based non-profit patient organization specialized in CML, based on specific medical and socio-economic criteria [51, 52]. Eligible patients are UC patients diagnosed with CML-Ph+ or CD117 positive GIST whose household incomes are less than 100,000 baht (US$3,225) per year. The administration of the program by independent third parties is intended to ensure independence in the evaluation and approval of patients. If approved, Novartis supplied imatinib though hospitals every three months based on requests from the treating physicians.

After negotiations with the Thai government to avert CL in January 2008, the company agreed to expand the GIPAP program and provided imatinib free of charge to all UC patients for the treatment of CML and GISTs. All patient applications are assessed by the Max Foundation and Novartis is responsible for drug distribution; in addition, Novartis also needs to share information about eligible patients with the NHSO for their records.

For SSS patients with CML-Ph+ who needed to pay out-of-pocket for imatinib at the time, the company had proposed in June 2003 a 50% price reduction to interested hospitals. It was unclear from public documents how many hospitals participated in the price negotiation. In July 2012, following negotiations between the company and the SWO, the 50% price reduction was extended to all SSS-contracted hospitals.

#### Utilization over time

Fig. 2(C) shows the utilization of imatinib over time. Since GIPAP implementation in 2003, imatinib use rose continually. Following expansion of the GIPAP program to all UC patients and licensing of alternative tyrosine kinase inhibitors, utilization of imatinib dropped briefly in the first half of 2009 but continued to increase and possibly more steeply following the 2012 price reduction for SSS patients.

Based on standard dosages of imatinib (Table 1), the median estimated number of patients treated increased significantly from 103 (95% CI 72–174) before to 350 (95% CI 307–398) after the 2009 implementation of the policies and programs.

### Nilotinib and Dasatinib (Non-NLEM)

#### Policy and program approaches

Nilotinib and dasatinib are second-generation tyrosine-kinase inhibitors for the treatment of CML-Ph+ and GISTs, similar to imatinib. They were approved for sale in Thailand in September and October 2008, respectively.

Pharmaceutical company initiated access program: The Tasigna International Patient Assistance Program (TIPAP) is a patient assistance program initiated in January 2009 by the manufacturer Novartis Co. Ltd. to facilitate access to nilotinib for CML-Ph+ and GIST patients who did not respond to or unable to tolerate imatinib and cannot afford the treatment.[18] Like GIPAP, the Max Foundation operates the TIPAP program for selected patients with financial problems defined as those with household incomes less than 100,000 baht (US$3,225 in May 2014) per year and covered by the UC scheme.

#### Utilization over time

Fig. 2(C) shows the utilization of nilotinib and dasatinib. After nilotinib and dasatinib were launched, sales increased over time, with volume of use per half-year per 100,000 persons of nilotinib higher than that of dasatinib during the study period. Since the products were launched, the estimated numbers of patients treated (based on standard daily doses in Table 1) were 39 (95% CI 24–60) and 20 (95% CI 10–25) for nilotinib and dasatinib, respectively.

## Discussion

In pluralistic health care financing systems, no single policy strategy can provide access to high-cost medicines for all who need them. Multi-pronged approaches are needed, implemented by multiple stakeholders who cooperate and negotiate within their realms of responsibilities and constraints. Our study described how government, payers, and pharmaceutical companies used various tools to increase access to targeted cancer medicines for different patient populations in Thailand. Not surprisingly, use of NLEM listed agents (letrozole and imatinib) was substantially higher than that of the unlisted drugs (trastuzumab, nilotinib, and dasatinib) for the same indications. However, stakeholders also implemented programs that made access possible for unlisted drugs in Thailand. Overall, various programs seem to have complemented each other in increasing use of the cancer medicines studied.

The goal of universal health coverage (UHC) is to ensure that all people can obtain the health services they need while protecting households from impoverishing out-of-pocket health spending.[56] One of the key factors that must be considered when implementing UHC is access to needed medicines.[56] Without access to medicines, countries cannot progress toward goals to reduce the number of avoidable deaths due to common conditions and increase survival of patients with severe diseases such as cancer. Along with preventing illness, ensuring equitable access to appropriate and affordable pharmacological treatment is crucial to achieving UHC and financial protection goals.

Thailand introduced UHC in 2002 and initiated a number of policies to achieve and sustain universal coverage implementation. To facilitate access to targeted cancer medicines, several stakeholders initiated different policies based on their authorities in the Thai health system. The Thai government ensured the availability of medicines by implementing the NLEM for all Thai people and payers applied the NLEM as the minimum reimbursement list for their enrollees. The Thai government then listed selected high-cost medicines in a new E2 subcategory of the NLEM, with a requirement of coverage over time, once financing for E2 category drugs was feasible. To make financing more feasible, the government and payers negotiated with companies to decrease prices.

To make medicines more affordable, payers pooled procurement and negotiated lower prices, saving hundreds of million baht annually.[57] Since January 2013, the SWO transferred its budget to NHSO to participate in pooled procurement and delivery of E2 medicines for SSS patients. Larger volumes, for more than 90% of total Thai population, are expected to increase negotiation power and to decrease purchasing prices further.

To make medicines more affordable, the Thai government also implemented highly controversial CL. To ensure the quality of generic medicines imported after the implementation of CL, the Thai Food Drug and Administrative (FDA) addressed Thailand’s drug qualification standards in the drug registration system, and implemented a post-import surveillance system and a drug quality reporting system.[50,58] An assessment of the impacts of CL suggested that importing less expensive generic medicines following CL resulted in savings in excess of US 140 million for the use of three cancer medicines over 5 years.[27] Although some had raised concerns about the negative effects of CL on trade, the study found no negative impacts on Thailand’s exports. Although CL constituted a legitimate strategy and seems to have benefitted the Thai health system, CL may not be a desirable policy strategy to improve access to medicines in every system: CL depends on national and international political circumstances, requires potentially confrontational actions, and is administratively cumbersome as it usually applies to one drug and one company at a time.

For non-listed NLEM medicines, pharmaceutical companies made medicines accessible to selected patients by introducing patient assistance programs and special marketing arrangements. An exemplary patient assistance program, GIPAP, was offered by Novartis in Thailand since 2003. Initially, this program was set up and managed by the company because imatinib was not listed on the Thai NLEM at that time. The program provided access to the drug for 1380 poor patients during the first five years of its implementation.[59] After the negotiations between the government and the pharmaceutical company, and to avoid CL, Novartis expanded the GIPAP program to cover all eligible UC patients since 2008. Following the successful negotiation and coverage expansion, the NLEM subcommittee listed imatinib on the 2008 NLEM. Although there is a trend in increased use of patient assistance programs, these programs tend to be heterogeneous, varying across hospitals, highly dependent on the continued support of manufacturers and visionary policy makers, and subject to the readiness of the delivery system to support provision of comprehensive care.[60] Implementing such programs efficiently on a large scale requires agreements among stakeholders, investments in health system infrastructure, and close monitoring so that eligible patients in need receive continued access to medicines.[61]

Under UHC expansion, expenditures are intended to shift from out-of-pocket (OOP) spending to public health expenditures.[62] Between 1995 and 2009, Thailand increased public health spending by 6.2 percent annually while out-of-pocket spending on health fell by 3.4 percent annually,[63] a development often cited as testimony to the successful achievement of UHC in Thailand.[64,65] Recently, the list of E2 medicines has been expanded and now includes 16 products (4 oncology medicines and 12 other essential medicines) indicated for 27 conditions.[2] Given the increasing needs for and availability of novel, usually very expensive treatments for cancer and other conditions, an important question concerns the financial sustainability of the health system. At a minimum, routine and careful monitoring of expenditures is needed to intervene as needed to ensure sustainability. In addition, other tools to increase access to novel medicines will likely be required in the context of limited financial resources, including risk-sharing schemes[66] and other public-private collaborations. Risk-sharing schemes are agreements between a payer and a pharmaceutical company in which the partners negotiate the price of a product and/or the overall spending depending on volumes sold, clinical outcomes achieved or patient populations who receive the drug.[67,68] The intent is that companies share the financial risk of payers to cover the drug, and pay for the drug when an agreed volume or budget is exceeded, or intended clinical outcomes are not achieved. Even broader public-private partnerships engage multiple actors to pursue long-term goals such as cancer control and health system strengthening.[69] Importantly, studies are needed to evaluate the impacts of expanding access to novel medicines on health outcomes for specific patient populations and the opportunity costs and health benefits achieved in the system as a whole.

Our study has a number of limitations. First, because interventions were multiple and in close proximity, we were unable to test whether discontinuities in market growth were due to specific policy changes using formal statistical methods such as interrupted time-series analysis. Instead, we estimated numbers of patients treated based on several conservative assumptions about use of the products in question for specific indications and at fixed recommended doses. To compare estimates, we applied the same assumptions in both pre- and post-policy implementation periods. Differences in estimated numbers of patients treated based on differences in product volumes sold could have occurred because of changes in therapeutic regimens over time, general market growth, or the complexities of supply systems and stock management. Nevertheless, visual inspection of market trajectories is a useful analytic tool. For example, we identified fluctuations of letrozole sales that were explained by pooled purchasing from GPO during 2010–2011. Second, we did not explore policies initiated by non-profit international organizations (i.e. donation programs). Such programs may provide targeted cancer therapies for specific patients. Nevertheless, our study captured policies that were initiated by key stakeholders at national and hospital levels. Third, given the lack of reliable epidemiologic and clinical data (including incidence of specific cancers, percentage of patients in each stage, percentage of patients receiving chemotherapy), we could not estimate the percentages of cancer patients in Thailand who would be eligible for the specific cancer therapies or the changes in patients receiving medicines among eligible patients. Fourth, because IMS Health sales data are estimates of national sales based on sampled hospital data, changes over time in relevant characteristics of hospitals—such as registration status with GPAP or whether a hospital negotiates prices with manufacturers—, compared to changes in composition of sample hospitals, may have biased our estimates of changes in imatinib sales. Lastly, given our use of IMS sales data, we were unable to assess whether access to the targeted therapies was equitable or whether the medicines were used appropriately. Individual-level data including patient demographic and clinical characteristics are needed to study equity in access and appropriateness of medication use. To do so, routine data should be collected to monitor and evaluate policies and programs for access to targeted cancer therapies, both from the perspectives of cancer care and overall health care in the system.

## Conclusions

Our findings highlight the interplay of policies initiated by different stakeholders to increase access to high-cost medicines. Although policy processes are likely to be highly context-specific, other countries may learn from the Thai experience. Key lessons include the importance of consistent political support and cooperation among health system stakeholders. No single approach is likely to facilitate access to targeted cancer therapies for all who need them; multiple approaches are needed that differ by stakeholder, by the nature of the target population, and by the regulatory status of each medicine. Continued research is needed to assess whether the utilization of expensive medicines is equitable, clinically appropriate and effective, and affordable at household and system levels.
